# A human inferred germline antibody binds to an immunodominant epitope and neutralizes Zika virus

**DOI:** 10.1371/journal.pntd.0005655

**Published:** 2017-06-12

**Authors:** Diogo M. Magnani, Cassia G. T. Silveira, Brandon C. Rosen, Michael J. Ricciardi, Núria Pedreño-Lopez, Martin J. Gutman, Varian K. Bailey, Helen S. Maxwell, Aline Domingues, Lucas Gonzalez-Nieto, Vivian I. Avelino-Silva, Mateus Trindade, Juliana Nogueira, Consuelo S. Oliveira, Alvino Maestri, Alvina Clara Felix, José Eduardo Levi, Mauricio L. Nogueira, Mauricio A. Martins, José M. Martinez-Navio, Sebastian P. Fuchs, Stephen S. Whitehead, Dennis R. Burton, Ronald C. Desrosiers, Esper G. Kallas, David I. Watkins

**Affiliations:** 1 Department of Pathology University of Miami, Miami, FL, United States of America; 2 Division of Clinical Immunology and Allergy, School of Medicine, University of São Paulo, São Paulo, SP, Brazil; 3 Hospital Sírio-Libanês, São Paulo, SP, Brazil; 4 Neurology Department, School of Medicine, University of São Paulo, São Paulo, SP, Brazil; 5 Instituto Adolfo Lutz, São Paulo, SP, Brazil; 6 Instituto Evandro Chagas, Belém, PA, Brazil; 7 Departamento de Moléstias Infecciosas e Parasitárias-(LIM-52), Instituto de Medicina Tropical de São Paulo e Faculdade de Medicina, Universidade de São Paulo, São Paulo, SP, Brazil; 8 Laboratório de Pesquisas em Virologia, Departamento de Doenças Dermatológicas, Infecciosas e Parasitárias, Faculdade de Medicina de São José do Rio Preto, São José do Rio Preto, SP, Brazil; 9 Laboratory of Infectious Diseases, National Institute of Allergy and Infectious Diseases, National Institutes of Health, Bethesda, MD, United States of America; 10 Department of Immunology and Microbiology, The Scripps Research Institute, La Jolla, CA, United States of America; 11 The Ragon Institute of Massachusetts General Hospital, Massachusetts Institute of Technology and Harvard University, Cambridge, MA, United States of America; University of North Carolina at Chapel Hill, UNITED STATES

## Abstract

The isolation of neutralizing monoclonal antibodies (nmAbs) against the Zika virus (ZIKV) might lead to novel preventative strategies for infections in at-risk individuals, primarily pregnant women. Here we describe the characterization of human mAbs from the plasmablasts of an acutely infected patient. One of the 18 mAbs had the unusual feature of binding to and neutralizing ZIKV despite not appearing to have been diversified by affinity maturation. This mAb neutralized ZIKV (Neut_50_ ~ 2 μg/ml) but did not react with any of the four dengue virus serotypes. Except for the expected junctional diversity created by the joining of the V-(D)-J genes, there was no deviation from immunoglobulin germline genes. This is a rare example of a human mAb with neutralizing activity in the absence of detectable somatic hypermutation. Importantly, binding of this mAb to ZIKV was specifically inhibited by human plasma from ZIKV-exposed individuals, suggesting that it may be of value in a diagnostic setting.

## Author summary

Antibody affinity maturation through somatic hypermutation (SHM) is thought to be critical for the development of antibodies with virus-neutralizing activity. Contrary to this notion, we describe novel human anti-Zika virus (ZIKV) antibodies with very low mutation levels, isolated from plasmablasts early after the onset of symptoms. Surprisingly, one IgG monoclonal antibody, P1F12, bound to ZIKV and neutralized the virus, despite having no detectable mutations. This antibody is specific for ZIKV and did not cross-react with DENV. Furthermore, plasma from ZIKV-positive individuals blocked the interaction of P1F12 with ZIKV, whereas plasma from DENV-positive patients did not have this inhibitory ability. P1F12 targets an immunodominant site, as ZIKV-positive samples blocked P1F12-ZIKV binding. Our study shows that isotype-switched virus-specific neutralizing Abs can develop in humans directly from germline sequences.

## Introduction

Zika virus (ZIKV) belongs to the genus *Flavivirus* of the *Flaviviridae* family and is related to dengue virus (DENV), yellow fever virus (YFV), Japanese encephalitis virus (JEV), and west Nile virus (WNV) [1]. The globally distributed mosquito species of the Aedes genus, vectors for many *Flavivirus*, can also transmit ZIKV [2, 3]. However, ZIKV remained a relatively minor and obscure cause of human disease for most of the second half of the 20^th^ century and was featured in a limited number of scientific reports. In fact, it was not until 2007 that autochthonous human infection was described outside Africa and continental Asia—in the Federated States of Micronesia [4–6]. Since then, reports from Brazil have chronicled a rapidly spreading epidemic that co-exists with DENV and chikungunya virus (CHIKV). The epidemic has spread north with mosquito-borne transmission being reported in many nations of the Americas as far north as Mexico and southern Florida [7–9]. More ominously, ZIKV has been implicated as the causative agent in fetal developmental problems [10, 11]. There are reports of ZIKV-associated birth defects, primarily brain abnormalities and microcephaly in infants born to mothers infected with ZIKV [12]. Virus has been recovered from amniotic fluid, placental, and brain tissues [13–21]. ZIKV infection has been classified as an ongoing threat by the World Health Organization. In the United States, the Centers for Disease Control and Prevention has issued guidance for the management of the infection in the general population, pregnant women, and infants [22–24]. Due to recent reports of sexually transmitted ZIKV infection, the CDC has also developed guidelines for prevention of this mode of transmission [22–27]. More recently, ZIKV transmission has also been described in Miami, Florida [28], suggesting that autochthonous spread could occur in any region of the U.S. inhabited by *Aedes spp*.

Treatment of a variety of human ailments using mAbs is revolutionizing our ability to ameliorate human suffering. For infectious disease, the Ebola epidemic highlighted the potential utility of a cocktail of three neutralizing (n)mAbs that block infection by the Ebola virus [29]. Most convincingly, the administration of a single nmAb up to five days post infectious virus exposure prevents the development of disease in Ebola-infected macaques [30]. Because mAbs can be engineered to prevent antibody-dependent enhancement by incorporating the L234A and L235A (LALA) mutations which reduce FcγR binding [31], they are a promising intervention in flaviviral therapies. Our long-term goal is to use a cocktail of LALA-mutated nmAbs to prevent ZIKV infection in at-risk individuals, primarily pregnant women.

Therapeutic nmAbs must be potent in order to be clinically viable, and most nmAb isolation strategies are based on the identification of high-titer, antigen-selected repertoires. Somatic hypermutation (SHM) in germinal center (GC) B cells provides the basis for selection of B cells producing Abs with increased affinity—a hallmark of the adaptive humoral response. This feature is conserved among mammals, highlighting the importance of Ab affinity enhancement for evolutionary fitness [32]. Thus, it is unsurprising that the vast majority of human Abs in the memory immunoglobulin (Ig)G pool have undergone affinity maturation and have, on average, 10–26 nucleotide substitutions from precursor genes [33]. The contribution of SHM to Ab-mediated viral neutralization is particularly clear for the chronically-induced broadly neutralizing antibodies to HIV [34–37]. Reversion of these anti-HIV nmAbs to precursor germline antibodies results in a drastic reduction or complete loss of viral neutralization [38–41]. Although mutated mAbs are found after secondary DENV infection, the role of these mutations in acute virus-neutralization and clearance is less clear [42–45]. Still, the prevalent thought is that antiviral Ab response involves the engagement of poor- or non-neutralizing germline clones generated by V(D)J rearrangement, followed by SHM-mediated refinement in germinal centers to enhance neutralization potency.

Here we describe the isolation of 18 plasmablast-derived human mAbs, sorted 12 days post onset of symptoms from a ZIKV-patient in São Paulo, Brazil. The patient reported a previous history of dengue infection and yellow fever vaccination (Table 1). A few of the isolated Abs neutralized ZIKV, most of them at relatively high concentrations. Interestingly, one of these mAbs (P1F12) exhibited no nucleotide mutations when compared to its corresponding germline sequences, but still recognized a ZIKV immunodominant epitope and neutralized the virus. These results suggest unforeseen roles for GC-independent responses against ZIKV and possibly other viruses.

**Table 1:** Patient details.

Identification				Diagnosis		Medical history	Initial Symptoms (D0)	Clinical history^a^	Time of Plasmablast Sort^a^
ID	City	Sex	Age	Urine	Blood				
533	São Paulo	F	56	PCR positive	PCR negative	dengue fever, YF vaccinated	Rash, Myalgia, Arthralgia	GBS initiation (D6), Hospitalized (D10), IVIG treated (D12).	D12

^a^Time point after onset of symptoms.

## Methods

### Human samples

Blood samples were collected from volunteer 533, a 56-year-old woman who reported a pruriginous skin rash that started six days prior to the beginning of acute neurological deficits suggestive of GBS. ZIKV infection was confirmed by a positive real-time reverse-transcriptase PCR assay for ZIKV RNA in urine samples collected at days 11 and 12 after the onset of the first rash symptoms. Blood and cerebrospinal fluid were negative for ZIKV RNA. Previous history of a single dengue infection and yellow fever immunization were also reported. Peripheral blood mononuclear cells (PBMCs) were obtained from blood samples collected 12 days post onset of symptoms. Blood samples from patient 533 were obtained after signing a written consent form approved by the University of São Paulo’s Institutional Review Board (CAPPesq 0652/09). Anonymized plasma samples from volunteers in Brazil and US were obtained from naïve and convalescent subjects with RT-PCR-confirmed ZIKV or DENV infection (S2 Table). Four volunteers donated samples post yellow fever vaccination.

### Flow cytometry and plasmablast sorting

We determined the frequency of plasmablasts in circulation by cytometric analysis of PBMCs obtained from blood collected in acid citrate dextrose (ACD) using a Ficoll-Paque (GE Lifesciences) gradient. Briefly, we stained fresh PBMC samples (1 x 10^6^ cells, room temperature, in the dark), with 100 μl of a cocktail containing the following fluorophore-antibody conjugates: phycoerythrin (PE)-CF594 anti-human CD3 (clone UCHT1; Becton Dickinson [BD]), PE-CF594 anti-human CD14 (clone MφP9; BD), Allophycocyanin (APC)-Cyanine (Cy)7 anti-human CD19 (clone SJ25C1; BD), Peridinin Chlorophyll Protein Complex (PerCP) anti-human CD20 (clone L27; BD), APC anti-human CD27 antibody (clone O323; Biolegend), Fluorescein isothiocyanate (FITC) anti-human CD38 (clone HB7; BD), PE anti-human CD138 (clone MI15, BD). We also included the fixable viability dye LIVE/DEAD® Fixable Red Dead Cell Stain Kit (Life Technologies) in the staining mix, in order to discriminate between live and dead cells. After 30 min, we washed the cells twice with FACS buffer (PBS, 0.5% FBS, 2 mM EDTA), resuspended with a PBS 1x solution, and stored at 4°C until acquisition on the same day. Samples were acquired using a BD FACSAria IIu flow cytometer and analyzed using FlowJo 9 (FlowJo). The plasmablast population was defined as live CD19+ CD3- CD14- CD20- CD27+ CD38+ cells (see gating and sort strategy in S1 Fig). Using this same plasmablast staining, fresh PBMC samples (5 x 10^6^ cells) were sorted on a BD FACSAria II flow cytometer. Single plasmablast cells were sorted into 96-well plates containing a lysis buffer designed to extract and preserve the RNA (250 mM Tris-HCl pH 8.3, 375 mM KCl, 15 mM MgCl_2_, 6.25 mM DTT, 250 ng/well yeast tRNA, Life Technologies; 20 U RNAse inhibitor, New England Biolabs [NEB]; 0.0625 μl/well IGEPAL CA-630, Sigma). After sorting, the RNA plates were immediately frozen in dry ice for subsequent cloning of the Ab chains.

### Ab repertoire analysis

We conducted reverse transcription followed by a nested PCR to amplify the variable region of the Immunoglobulin (Ig) chains using described protocols with minor modifications [46]. Briefly, cDNA was synthesized in a 25 μl reaction using the original sort plates. Each reaction contained 1 μl of 150 ng random hexamer (IDT), 2 μl of 10 mM dNTP (Life Technologies), 1 μl of SuperScript III Reverse Transcriptase (Life technologies), 1 μl molecular biology grade water, and 20 μl of single sorted cell sample in lysis buffer (described above). The reverse transcription reaction was performed at 42 ˚C for 10 min, 25 ˚C for 10 min, 50 ˚C for 60 min, 94 ˚C for 10 min. After the reaction was completed, cDNA was stored at -20 ˚C. Heavy and light chains were amplified in three different nested PCR reactions, using a mix of 5’ V-specific primers with matching 3’ primers to the constant regions of IgG, IgL, and IgK. PCR reactions were conducted using HotStarTaq Plus DNA Polymerase (Qiagen). The second set of PCR reactions was carried out with primers redesigned to incorporate restriction sites compatible with subcloning into rhesus IgG1 expression vectors, instead of the original human vectors [46]. We sequenced the amplified and cloned products using primers complementary to the Ig constant regions. Sequences were analyzed using IgBLAST and IMGT/V-QUEST to identify V (D) J gene rearrangements, as well as SHM levels [47, 48].

### Ab expression and purification

We expressed mAbs in Expi293F (ThermoFisher) human cell lines. The plasmids encoding heavy and light chains were co-transfected using the ExpiFectamine 293 Transfection kit (A14525, ThermoFisher). After 5–6 days, we harvested the secreted mAb in the supernatant. Ig concentration in the supernatant was determined by an anti-rhesus IgG ELISA, before we proceeded with the functional assays. For the experiments with purified mAbs, we used Protein A Plus (Pierce)-containing columns to remove the impurities. The concentration of purified protein was determined by measurement of absorbance at 280 nm (NanoDrop, Thermo Scientific).

### Virus capture assay and recombinant E protein ELISA

P1F12 binding was determined by both virus capture assay (VCA) and recombinant (r)E ELISAs. The VCA plates were coated overnight with the mouse-anti-*Flavivirus* monoclonal antibody 4G2 (clone D1-4G2-4-15, EMD Millipore) followed by incubation with viral stocks (ZIKV or DENV). The rE ELISA plates were coated with ZIKV E Protein (MyBiosource, MBS596001) diluted to 5 μg / ml in PBS. After the coating step, the plates were washed with PBS and mAb samples diluted to 1 μg / ml were added to designated wells and incubated for 1 h at 37 ˚C. Subsequently, the plates were washed and detection was carried out using a goat anti-human IgG HRP secondary Ab (Southern Biotech), which was added to all wells at a dilution of 1:10,000. Following a 1 h incubation at 37C, the plates were washed and developed with TMB substrate at room temperature for 3–4 min. The plates were developed with TMB substrate at room temperature for 3–4 min. The reaction was stopped with TMB solution and absorbance was read at 450 nm.

### Flow cytometry-based neutralization assay

The neutralizing potency of the mAbs was measured using a flow cytometry-based assay [49, 50]. In brief, recombinant mAbs (transfection supernatant or purified) were diluted and pre-incubated with ZIKV (Paraiba) or the reference DENV serotypes in a final volume of 220 μL for 1 h at 37 ˚C. The virus and mAb mixture (100 μL) was added onto wells of a 24-well plate of 100% confluent Vero cell monolayers in duplicate. A new seed of Vero cells (CCL-81TM) was obtained from the American Type Culture Collection (ATCC) repository for this study. The inoculum was incubated in a 37 ˚C incubator at 5% CO_2_ for one hour with agitation of the plates every 15 min. After one hour, the virus and mAb-containing supernatants were aspirated and the wells were washed with media. Fresh media was then added and the plates were incubated for a total of 24 hours. Cells were trypsinized with 0.5% trypsin (Life Technologies), fixed (BD cytofix), and permeabilized (BD cytoperm). Viral infection was detected with the 4G2 antibody (Millipore) recognizing ZIKV or DENV, followed by staining with an anti-mouse IgG2a APC fluorophore-conjugated secondary reagent (Biolegend). The concentration to achieve half-maximal neutralization (Neut_50_) was calculated using a nonlinear regression analysis with Prism 7.0 software (GraphPad Software, Inc.). The following strains were used in our neutralization assays: ZIKV Paraiba 2015 (KX280026.1), DENV1-West Pac (U88535.1), DENV2-NGC (AF038403.1), DENV3-Sleman/78 (AY648961), and DENV4-Dominica (AF326573.1)

### Plaque reduction neutralization test (PRNT)

PRNTs were conducted as previously described [51]. Briefly, purified P1F12 was serially diluted in OptiMEM supplemented with 2% human serum albumin (VWR), 2% fetal bovine serum, and gentamicin. ZIKV Paraiba 2015 was diluted to a final concentration of ~500–1000 PFU / mL in the same diluent added to equal volumes of the diluted Ab. The virus/mAb mixture was incubated at 37 ˚C for 30 min. Cell culture medium was removed from 90% confluent monolayer cultures of Vero cells on 24-well plates and 100 μl of the virus/Ab mixture was transferred onto duplicate cell monolayers. Cell monolayers were incubated for 60 min at 37 ˚C and overlaid with 1% methylcellulose in OptiMEM supplemented with 2% FBS 2mM glutamine + 50 μg / ml gentamicin. Samples were incubated at 37 ˚C for four days after which plaques were visualized by immunoperoxidase staining, and a 50% plaque-reduction neutralization titer was calculated.

### P1F12-ZIKV binding inhibition assay

Inhibition of P1F12 mAb binding was determined by ELISA. To begin, the ELISA plate was coated with mouse anti-*Flavivirus* monoclonal antibody 4G2 (EMD Millipore) diluted 1:1,000 in carbonate binding buffer and incubated overnight at 4 ˚C. The next day, the plate was washed five times with PBS-Tween20 and wells were blocked with 5% skim milk in PBS for 1h at 37 ˚C. After the block step, the plate was washed and virus samples were added to designated wells for 1h incubation at room temperature. Subsequently, the plate was washed with PBS only and corresponding blocking plasma samples were added for 1h at 37 ˚C. Following the plasma block, the plate was washed and P1F12 was added to corresponding wells for 1 h at 37 ˚C. P1F12 was detected using a rhesus IgG-specific antibody (mouse anti-monkey IgG-HRP clone SB108a; Southern Biotech). Thereafter, the plate was washed and wells were developed with TMB substrate at room temperature for 3–5 min before the reaction was stopped with TMB Stop Solution. Absorbance was determined at 450 nm.

## Results

### Patient

We isolated plasmablasts from patient 533 who presented with suspected Guillain-Barré syndrome (GBS) (Table 1) (first day of symptoms = D0). The patient had a previous history of dengue infection and yellow fever vaccination (Table 1). The previously healthy 56-year-old woman presented to the emergency room (D6) reporting a progressive paresthesia mainly in the extremities of her hands, along with acute, intermittent pain in her left forearm during the previous four days. At physical examination, the patient presented with a grade IV asymmetric muscular weakness and hypoesthesia in her left limbs, with abolished deep tendon reflexes in the lower limbs. A mild weakness of her left facial muscles was also noted. The patient reported no respiratory disorders and no hoarseness, and no signs of dysautonomia were detected at the clinical evaluation. Fever, conjunctivitis, and myalgia or joint pain were absent during the illness. Afterwards, the patient was hospitalized with a clinical diagnosis of GBS, for which an intravenous human Ig (IVIG) treatment was initiated at a dosage of 0.4 g / kg / day for 5 days. Cerebrospinal fluid analysis and an electroneuromyogram were performed on fourth (D10) and fifth (D11) days after neurological symptom onset, respectively; the results were within normal limits. The electroneuromyogram was repeated on the 15^th^ day of neurological symptoms, but no significant abnormalities were noted despite the persisting weakness in the patient’s left leg and arm. During the treatment with IVIG, the patient presented with transient worsening of her hemiparesis, but progressively recovered over the course of weeks after discharge from the hospital. At 32 days post-neurological symptom onset (D38), a physical exam revealed significant improvement of muscular strength and abolished deep tendon reflexes in the lower limbs. The remittent skin rash cleared completely 10 days after its initial emergence.

Blood, cerebrospinal fluid and urine samples were collected on the 5^th^ day of neurological symptoms (D11) for detection of ZIKV by RT-PCR. The urine sample was ZIKV-positive by PCR, while blood and cerebrospinal fluid were negative. A saliva sample collected on D15 was negative for ZIKV.

### Isolation, binding, and neutralization testing of mAbs

**Fig 1:**
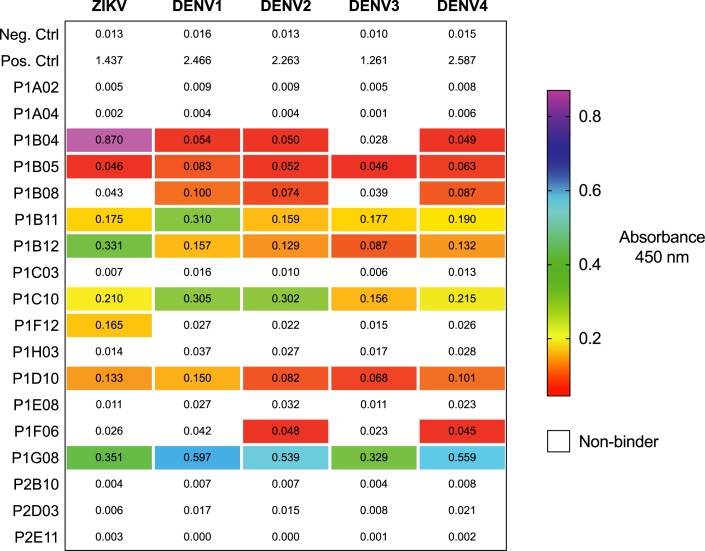
P1F12 mAb binds to whole ZIKV, but not DENV. The extent of mAb binding to ZIKV and the four DENV serotypes was quantified using a virus capture ELISA. The ability of purified mAbs (1 μg / ml) to bind to captured DENV and ZIKV was assessed. Absorbance (Abs 450) values higher than three times the negative control wells were considered binders.

**Fig 2:**
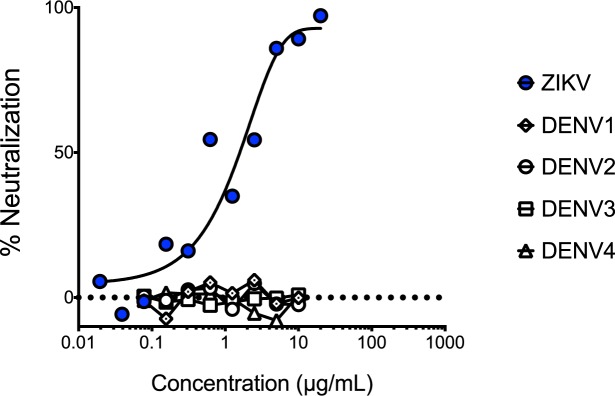
P1F12 mAb neutralizes ZIKV. P1F12 neutralization curves are presented as the reduction of Vero-cell infectivity measured by flow cytometry.

We isolated plasmablasts from peripheral blood mononuclear cells (PBMCs) collected on day 12 (Table 1). From wells containing single-sorted cells, we amplified, cloned, and sequenced heavy and light Ab chains using 5’ primers complementary to the V gene segments and a 3’ primer annealing to the constant IgG region [46]. This resulted in 18 paired heavy and light chains (S1 Table). Eight of the 18 mAbs bound to ZIKV (Fig 1). Seven of these mAbs exhibited cross-reactivity to one or more of the DENV serotypes, and a single mAb–P1F12–bound exclusively to ZIKV. Interestingly, two mAbs bound to DENV but not ZIKV. We tested the neutralization potency of the ZIKV-specific P1F12 mAb in a flow-based neutralization assay and a plaque reduction neutralization test (PRNT) and found that it neutralized ZIKV at approximately 2 μg / ml (PRNT_50_) (Fig 2).

### Unusual sequence of P1F12

**Fig 3:**
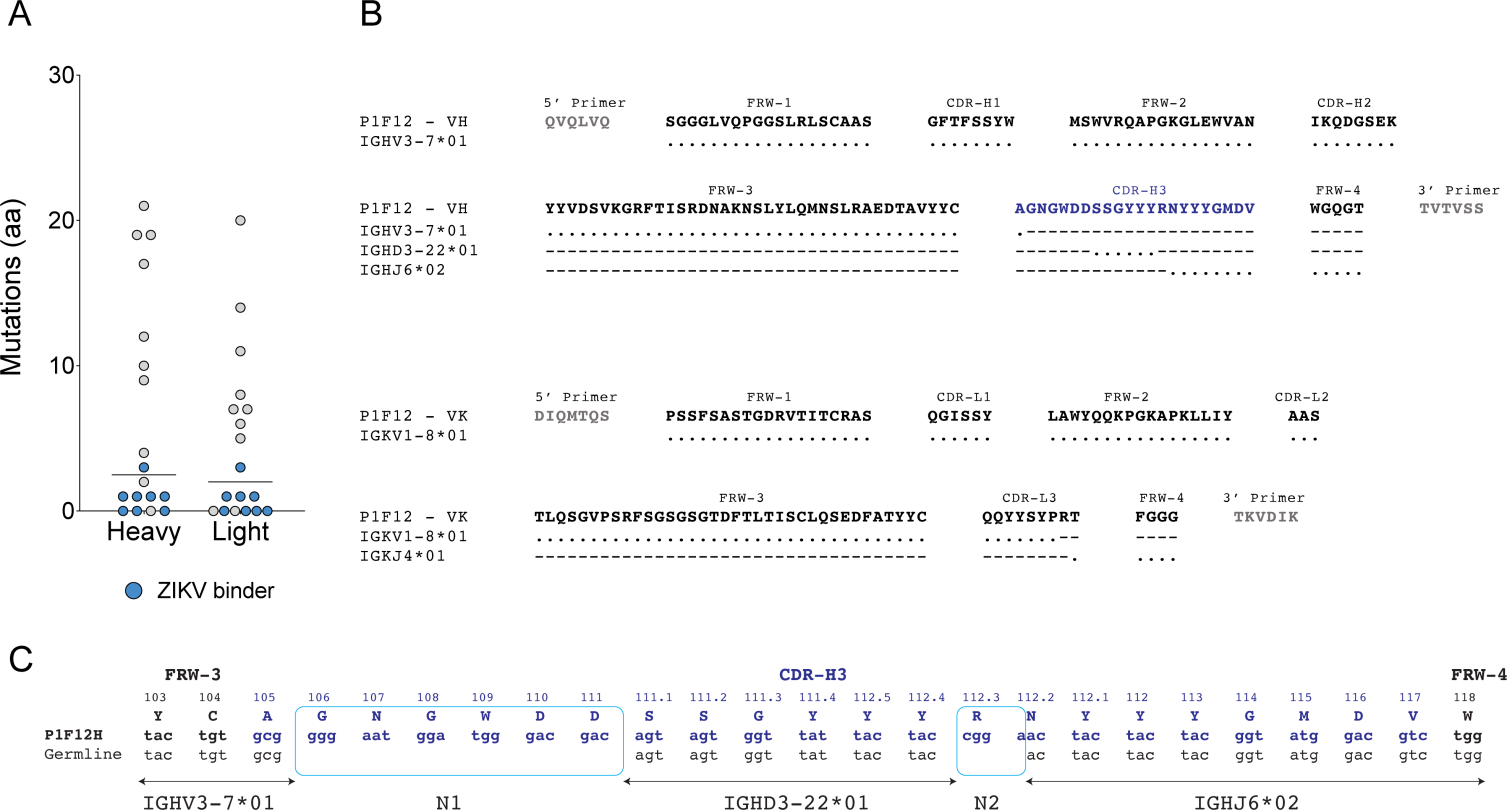
Plasmablast-derived ZIKV-specific mAbs had low SHM levels. A) Number of amino acid mutations from heavy and light chain germline sequences. B) Amino acid alignment of P1F12 to germline genes shows no mutations downstream of cloning primer. Dots “.” indicate sequence identity to the germline gene (shown in each row). Dashes “-” indicate that the Ab does not align to the annotated germline gene sequence on that position. The CDR-H3 sequence is indicated in blue. C) Nucleotide alignment of P1F12 CDR-H3 junction to germline genes. Boxes indicate junctional diversity between V and D (N1), and D and J (N2) gene segments. Framework (FWR) and complementarity-determining regions (CDRs) boundaries are directly annotated on top of the Ab sequence. Antibody regions were determined using IMGT/V-QUEST.

Analysis of the isolated antibody variable (V) domain sequences revealed five mAbs with average gene mutation levels (10–26 nucleotide modifications), two mAbs with over 30 nucleotide substitutions, and 11 mAbs with unusually low levels of SHM for isotype-switched mAbs (lower than 10 changes) (S1 Table). The most highly mutated mAbs (P1B08 and P1C03) were not ZIKV-specific by binding (S1 Table). In fact, the eight ZIKV-binding mAbs had the lowest SHM levels, including four mAbs lacking clearly recognizable mutations when compared with putative heavy and light chain germline precursors (S1 Table, Fig 3). Except for junctional diversity, the ZIKV-neutralizer P1F12 mAb heavy chain did not exhibit signs of antigen-selected Ig diversification. P1F12 had an identical sequence to the Ig heavy chain variable (IGHV) genes segment IGHV3-7*01 up to the amino acid C105, prior to the CDR-H3 (International Immunogenetics Information System [IMGT]) [52]. However, position G106–the site of the junction between IGHV and the IGH diversity (IGHD) genes–differed from the germline reference. Interestingly, this region is part of a segment (N1) with non-germline nucleotides corresponding to six amino acids identified between the IGHV and IGHD genes (Fig 3C). This segment is likely the result of N nucleotide additions generated during B cell Ig gene rearrangement, prior to antigen selection. Because of the lack of mutations elsewhere in the sequences, it is likely that the R106G substitution was also generated during this developmental step. The downstream sequence corresponding to the junction between IGHD3-22*01 and the IGH joining (IGHJ) IGHJ6*02 genes also revealed similar nucleotide insertions. Likewise, the Kappa (K) chain junction between the IGKV1-8*01 and IGKJ4*01 genes also contained one insertion. Although we cannot rule out the possibility of SHM-mediated nucleotide changes in the N insertion regions, no mutation was identified in the remainder of the regions of the heavy and light chains. Thus, the P1F12 mAb is likely very close or identical to the original V-(D)-J gene rearrangement in the naïve B cell before antigen contact.

### P1F12 recognizes an immunodominant epitope on ZIKV

**Fig 4:**
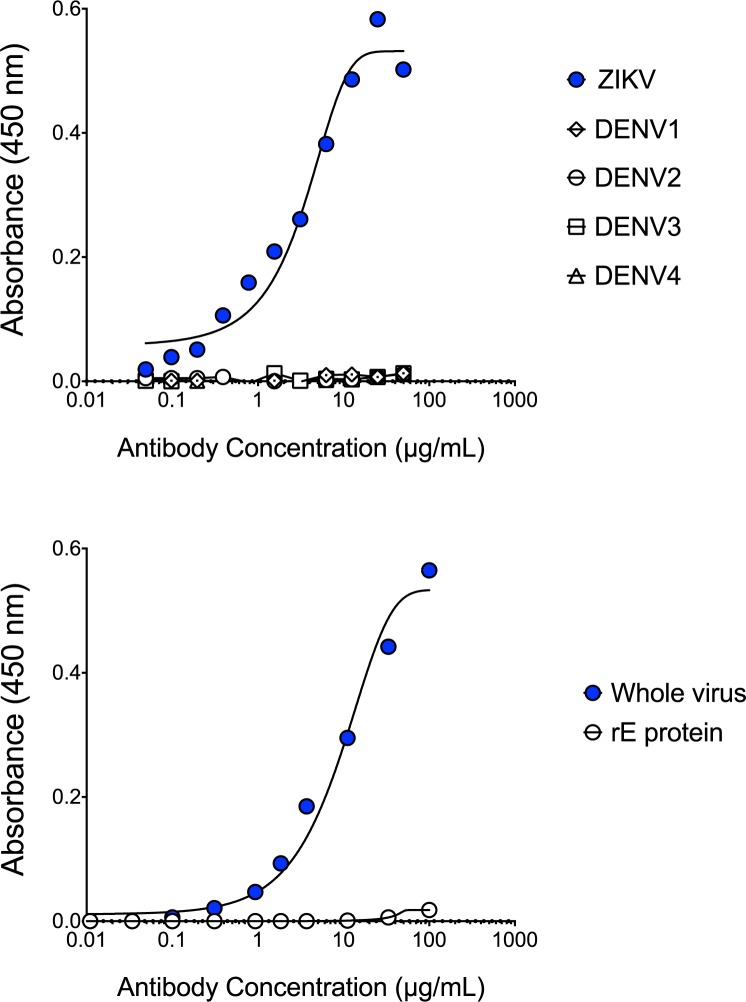
P1F12 mAb binds to whole ZIKV, but not to DENV or recombinant ZIKV E protein. P1F12 binding determined by both Virus Capture ELISA (top panel) and recombinant E protein ELISA (bottom panel) (19kDa protein without hydrophobic region). Control Absorbances: Whole Virus—Hu0004 (ZIKV+): 2.017, Hu002 (ZIKV-): 0.046. Control Absorbances rE: Whole Virus—Hu0004 (ZIKV+): 2.006, Hu002 (ZIKV-): 0.033.

**Fig 5:**
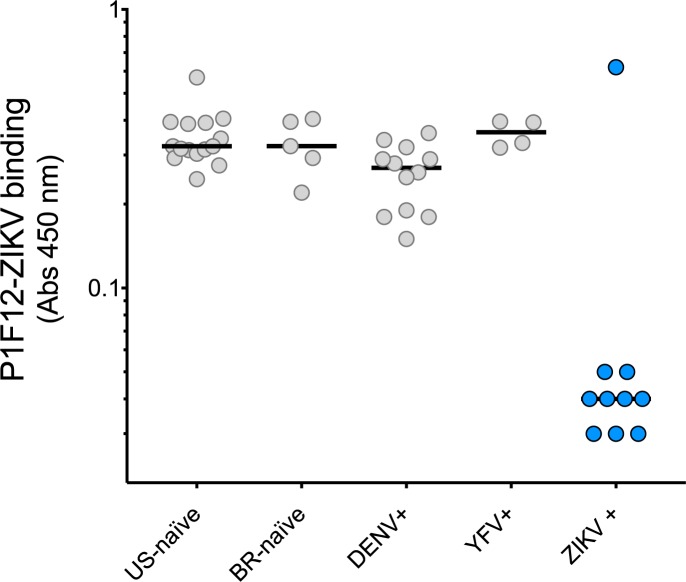
Inhibition of ZIKV-P1F12 binding discriminates plasma from ZIKV and DENV exposures. A modified virus capture ELISA was conducted to assess the ability of plasma from 46 individuals to block the binding of P1F12 to whole ZIKV. Captured ZIKV was incubated with 1/10 diluted plasma from naïve (US and Brazil) and DENV+, YFV+ or ZIKV+ volunteers prior to addition of purified P1F12. Viral infection was determined by RT-PCR. ZIKV-bound P1F12 was detected using an HRP-conjugated secondary Ab specific for the rhesus IgG1 Fc region of recombinant P1F12. ZIKV+ (blue circles), but not ZIKV- (gray circles) plasma inhibited binding of P1F12 mAb to ZIKV.

To investigate whether P1F12 recognizes an immunodominant ZIKV epitope, we used a serological blocking assay. In brief, this assay detects the presence of competing Abs that can inhibit the P1F12 mAb binding to its epitope. Because P1F12 did not bind to recombinant E protein (Fig 4) we used whole virus in our binding assays. We captured ZIKV on the plate using the 4G2 mAb (pan-*Flavivirus*), and incubated ZIKV with plasma from patients with diverse histories of DENV and ZIKV exposure (S2 Table). We added unlabeled P1F12 (engineered with rhesus IgG1 constant regions) and detected binding of the mAb using a HRP-labeled mouse anti-rhesus mAb (Fig 5). Nine of ten plasma samples from individuals that had been infected with ZIKV blocked the binding of P1F12 in a blinded test (Fig 5, S2 Table). Similar blocking activity was observed regardless of whether individuals had been previously infected with DENV or had been vaccinated for yellow fever. In contrast, little or no blocking activity was observed by DENV+ plasma in the absence of prior ZIKV exposure (Fig 5). Furthermore, this recognition was specific in that it was not observed in 14 of 14 DENV-only infected individuals. Thus, the P1F12 serological blocking assay accurately predicted previous ZIKV exposure, as confirmed by RT-PCR, in all but one of the patient plasma samples tested. Although this patient, donor 1302, had a positive urine RT-PCR result for ZIKV, plasma from 1302 did not block P1F12 binding to ZIKV (S2 Table). Interestingly, the plasma did not exhibit detectable ZIKV-neutralizing activity, suggesting that this patient did not mount a measurable antibody response against ZIKV. In conclusion, only the plasma that inhibited ZIKV infection of Vero cells contained P1F12-blocking antibodies.

## Discussion

Here we show that a IgG mAb with no detectable SHM was generated against ZIKV early in infection. Remarkably, despite being germline-encoded, this mAb is ZIKV-specific and does not bind to any of the four DENV serotypes. Furthermore, this mAb not only neutralizes ZIKV, but it also binds to an immunodominant epitope on the virus. Remarkably, despite being germline-encoded, P1F12 binds specifically to ZIKV and does not cross-react with any of the four DENV serotypes. Our results also suggest that P1F12 recognizes a unique epitope on ZIKV. It is unclear how this Ab developed such specificity without SHM. Finally, these findings suggest that affinity maturation is not necessary for the generation of isotype switched virus-neutralizing Abs.

Low levels of SHM in Abs possessing neutralizing activity have been previously reported in mice and humans [53–55], supporting the idea that germline-encoded mAbs can indeed neutralize. Abs with low levels of SHM have also been reported during the acute phase of human DENV infection, but it was not clear that these Abs contributed to the antiviral neutralization activity [56]. In studies in mice, VSV-specific mAbs lacking SHM have been isolated previously [53]. Interestingly, secondary, but not primary, mouse Abs against VSV had mutations [57]. Furthermore, the reversion of these mutated Abs to non-mutated precursors reduced, but did not abrogate, VSV binding and neutralizing activity. The binding differences between the mutated and germline Abs were much less pronounced than might be expected [57]. Additionally, mice that cannot conduct SHM due to AID knockout still mounted neutralizing Ab responses against Friend virus, a strain of murine leukemia virus [55]. It has been suggested that these Abs lacking extensive SHM undergo a GC-independent developmental pathway [58], although the mechanistic basis for this phenomenon remains to be elucidated.

Rapid, GC-independent responses might be particularly relevant in the control of acute cytopathic viruses [55, 58, 59]. The GC-independent Abs would arise quickly after infection and then curtail viral replication, preventing virus-mediated damage [60]. Even more provocatively, Hangartner *et al*. have argued that cytopathic viruses specifically evolved to retain binding to these germline sequences to decrease host lethality and increase fitness. On the other hand, chronic viruses may have evolved to avoid germline-binding and development of neutralizing responses to persist [60]. So far, these hypotheses remain unsubstantiated by the lack of evidence for strictly germline neutralizing Ab responses in humans. While our experiments were not specifically designed to detect GC-independent responses, it seems likely that the isotype-switched P1F12 originated directly from a germline precursor.

We isolated P1F12 from a ZIKV-infected individual that developed neurological complications compatible with GBS and was treated with IVIG. Underlying factors that influence the potential association of GBS and ZIKV infection might involve an autoimmune process, which could influence the development of immune responses [61]. Additionally, IVIG may have had a role in the selection of the Ab responses mounted by peripheral B cell repertoires [62]. This is unlikely, however, since the patient initiated IVIG treatment on the same day that the plasmablasts were isolated. It is possible, then, that GBS or IVIG-treatment influenced the development of P1F12. These potential associations are difficult to determine and were outside the scope of this study. It is clear, however, that these responses were not exclusive to volunteer 533, as P1F12 binding can be blocked by the serum of most ZIKV-infected individuals (Fig 5).

Recently described ZIKV-specific mAbs derived from Epstein-Barr virus–immortalized memory B cells are highly polyclonal and have undergone SHM [42]. However, SHM levels in these human anti-ZIKV mAbs were lower than SHM levels in mAbs isolated in response to primary infections or vaccination (SARS- CoV, H5N1, rabies vaccine), recurrent or chronic infections (RSV, PIV, *Staphylococcus aureus*, *Klebsiella pneumoniae*, HCMV, HCV) or autoimmune diseases [42]. Wang *et al*. have recently reported the isolation of 13 new ZIKV-specific mAbs from memory B cells, three of which had very little SHM [45]. These mAbs were isolated from memory cells sorted with soluble and monomeric ZIKV E proteins and, in contrast to P1F12, bind to the recombinant protein [45]. In contrast, we isolated ZIKV-specific mAbs from circulating plasmablasts at D12. The peak recall of memory B-cell derived plasmablasts is thought to occur within the first week post-secondary infection [63, 64]. Thus, it is probable that most of the isolated mAbs did not have a memory-B cell origin, and it remains possible that some of the plasmablasts were sorted from the basal population that circulate in low frequencies in the blood. In conclusion, the isolation of mAbs using different B cell methods suggest that anti-ZIKV mAbs with germline characteristics are not limited to specific B cell subtypes [42, 45]. Notably, the anti-ZIKV mAbs isolated to date are less mutated than the mAbs isolated after related DENV infections [42–45]. Together, these findings suggest possible differences in the development of Ab responses against ZIKV.

Unfortunately, despite our efforts, we were unable to map P1F12’s binding site. We first employed an *in vitro* escape assay [65], which did not result in a single mutated consensus sequence. Also, P1F12 did not bind to the prM/E proteins expressed in cells, precluding our ability to map this interaction using an Ala-mutated envelope panel [66, 67]. Characterizing this interaction will, thus, require a significant effort that is beyond the scope of the current manuscript. Because the P1F12 mAb retains the ability to bind virions, our conclusion is that it binds to a conformational epitope.

Based on the cohort of human plasma samples tested in this study, it appears that most ZIKV-infected individuals mount Ab responses against the epitope recognized by P1F12. This epitope is recognized by Abs in individuals previously infected by ZIKV, thereby preventing the binding of P1F12. By contrast, Abs in the plasma from individuals previously infected by any of the DENV serotypes, do not prevent binding of P1F12. P1F12 may, therefore have potential as a diagnostic. Several diagnostic options for testing for ZIKV exposure exist, including RT-PCR, IgM ELISA, and PRNT methods [22, 68]. While it is relatively straightforward to detect ZIKV nucleic acid during the acute phase in blood, urine, saliva, and semen, it has proven more difficult to design rapid and effective diagnostics for ZIKV exposure in the chronic phase. For samples collected after the first week of symptoms, the initial test is an anti-ZIKV, anti-DENV, anti-CHIKV virus IgM ELISA [68]. However, in patients who have received a flaviviral vaccine (DENV, YFV, or JEV) and/or have been infected with any Flaviviruses in the past, these assays may be difficult to interpret due to the cross-reactivity of the Abs [68–73]. Thus, a positive IgM test needs to be confirmed with a laborious PRNT assay. IgM antibodies persist for 2–12 weeks in serum, and sera from individuals previously infected for more than 12 weeks would also have to be confirmed with a virus neutralization-based method [68]. Our plasma inhibition assay may, perhaps, provide an alternative to these other techniques.

In this study, we isolated plasmablast-derived Abs from a ZIKV-infected individual with unusual characteristics. The human IgG P1F12 has no or limited SHM yet binds to an immunodominant ZIKV epitope that is not present on any of the four DENV serotypes. Furthermore, this mAb can neutralize the virus with a Neut_50_ of approximately 2 μg / ml. Our results suggest that SHM-independent pathways may generate neutralizing Abs in the responses against ZIKV.

## Supporting information

S1 Fig Plasmablast gating and sort strategy. (EPS)

S1 Table Gene usage and SHM levels in the plasmablast-derived mAbs. (PDF)

S2 Table Plasma samples. (PDF)
