# Supplementary material for: A human inferred germline antibody binds to an immunodominant epitope and neutralizes Zika virus
Source: PLoS Negl Trop Dis. 2017 Jun 12;11(6):e0005655. doi: 10.1371/journal.pntd.0005655 (PMC5481143; doi:10.1371/journal.pntd.0005655)
Supplement: S1 Table — (PDF) [file pntd.0005655.s002.pdf]

S1 Table. Gene usage and SHM levels in the plasmablast-derived mAbs.

mAb ID	ZIKV binding (Abs 450nm)	Human heavy chain V gene	Divergence (aa) from germline	CDRH3	Human light chain V gene	Divergence (aa) from germline	CDRL3
P1A02	0.01	IGHV5-51*01	4	CARHQPQGDTASHGMDVW	IGKV1-39*01	7	CQQSFNRLWTF
P1A04	0.00	IGHV1-18*01	10	CTKIDLHWDGVNGYDVSYFENW	IGKV1-27*01	5	CQKYHSAPWTF
P1B04	0.87	IGHV4-4*07	1	CARESGSLYMDVW	IGKV3-11*01	1	CQQRSNWPLTF
P1B05	0.05	IGHV3-23*04	1	CAKGMYYDFWSGSNDAFDIW	IGKV1-39*01	1	CQQSYSTPRTF
P1B08	0.04	IGHV4-39*07	21	CARTGSRRWYGMDVW	IGKV3-20*01	14	CQQYGSSVWAF
P1B11	0.18	IGHV3-23*04	1	CAKSFYRDFWSGSNDAFDIW	IGKV1-39*01	1	CQQSYSTPRTF
P1B12	0.33	IGHV4-34*01	0	CARRGGRCSSTSCYPYYYYYYMDVW	IGKV3-20*01	0	CQQYGSSPPKLTF
P1C03	0.01	IGHV4-4*07	19	CAKGGVTPGGGTSGTWFNPW	IGKV3-15*01	11	CQQYDHWPPYTF
P1C10	0.21	IGHV3-23*04	0	CAKSFYRDFWSGSNDAFDIW	IGKV1-39*01	0	CQQSYSTPRTF
P1F12	0.17	IGHV3-7*01	0	CAGNGWDDSSGYYYRNYYYGMDVW	IGKV1-8*01	0	CQQYYSYPRTF
P1H03	0.01	IGHV4-59*08	12	CVRHEIAGTTGAFDIW	IGKV4-1*01	6	CQQYYSIPWTF
P1D10	0.13	IGHV3-21*01	1	CARGRYGYSYALDYW	IGLV6-57*02	3	CQSYDSSNHVVF
P1E08	0.01	IGHV4-39*07	19	CARKAGYYYDYW	IGLV3-1*01	20	CQTGDTTTVF
P1F06	0.03	IGHV3-21*01	0	CARDFRGGYYGSGDAFDIW	IGLV1-44*01	0	CAAWDDSLNGSYVF
P1G08	0.35	IGHV4-34*01	3	CARKRWQQLRRNWFDPW	IGLV1-51*01	0	CGTWDSSLSAVVF
P2B10	0.00	IGHV3-23*04	9	CAKVRRVVVIVHDAFDVW	IGKV1-16*02	7	CQQYSFYPLTF
P2D03	0.01	IGHV3-23*04	17	CAKVVGLRLDDAFDIW	IGKV1-5*03	8	CQQYLSYCTF
P2E11	0.00	IGHV3-23*04	2	CAKDRGYYDSSGYYYSEAFDYW	IGKV3-11*01	0	CQQRSNWSGITF
