# Supplementary material for: A human inferred germline antibody binds to an immunodominant epitope and neutralizes Zika virus
Source: PLoS Negl Trop Dis. 2017 Jun 12;11(6):e0005655. doi: 10.1371/journal.pntd.0005655 (PMC5481143; doi:10.1371/journal.pntd.0005655)
Supplement: S2 Table — (PDF) [file pntd.0005655.s003.pdf]

Sample[a]	Donors ID	Sex	Age	Previous dengue[b]	Previous YF immunization[b]	Days post most recent exposure[c]	P1F12-ZIKV inhibition assay (Abs_{450})
ZIKV+	hu0004[d]	F	26	No	No	46	0.04
	1302[e]	M	35	No	Yes	110	0.62
	801	F	53	Yes	Yes	19	0.04
	802	F	29	No	Yes	19	0.05
	533	F	56	Yes	Yes	159	0.03
	3002	F	51	No	Unk	19	0.04
	3004	F	59	Yes	Unk	19	0.03
	3012	M	60	Yes	Unk	17	0.05
	3013	F	72	Yes	Unk	16	0.03
	3018	F	43	No	Unk	15	0.04
DENV+[f]	138	F	56	Unk	No	33	0.34
	152	M	87	No	Yes	30	0.15
	168	F	32	No	No	32	0.32
	177	M	21	No	No	29	0.18
	184	M	23	No	No	29	0.26
	507	F	47	No	No	33	0.28
	1235	M	17	No	No	30	0.18
	1208	F	33	No	Unk	30	0.25
	1248	M	30	No	Unk	31	0.36
	1252	F	43	No	Yes	31	0.19
	1280	F	38	No	No	34	0.29
	1294	F	28	No	No	30	0.29
YF vaccine	FA2002	M	34	Unk	Yes	28	0.319
	FA2004	F	43	No	Yes	28	0.393
	FA2005	M	30	Unk	Yes	28	0.3315
	FA7006	M	73	Unk	Yes	28	0.396
Brazilian naïve	12	F	27	Yes	Yes	N/A	0.22
	515	F	18	No	Yes	N/A	0.293
	1255	F	18	No	Yes	N/A	0.323
	1261	F	15	Unk	Unk	N/A	0.4045
	1273	M	62	No	Yes	N/A	0.395
U.S. naïve	hu0002	M	28	No	No	N/A	0.57
	Class 1	F	20	No	No	N/A	0.3225
	Class 2	F	19	No	No	N/A	0.3125
	Class 3	F	23	No	No	N/A	0.4055
	Class 4	F	18	No	No	N/A	0.392
	Class 5	F	19	No	No	N/A	0.3885
	Class 6	F	21	No	No	N/A	0.2925
	Class 7	F	22	No	No	N/A	0.246
	Class 8	M	20	No	No	N/A	0.3035
	Class 9	M	19	No	No	N/A	0.316
	Class 10	M	20	No	No	N/A	0.3145
	Class 11	M	21	No	No	N/A	0.3945
	Class 12	F	27	No	No	N/A	0.344
	Class 13	F	24	No	No	N/A	0.2755
	Class 14	M	24	No	No	N/A	0.3225

[a] ZIKV and DENV samples determined by RT-PCR; [b]Donor reported; [c]Days after the onset of symptoms or YF immunization; Unk, unknown; N/A, Not applicable. [d]Patient was not ZIKV RT-PCR positive, but had traveled to a ZIKV endemic area during the outbreak, had symptoms that matched ZIKV infection, and was found to have a plasma $Neut_{50}$ titer of 1:7,211 against ZIKV at time point collected. [e]Patient urine was positive by RT-PCR, but patient had no ZIKV-neutralizing serum activity in the sample tested. [f]All donors were positive for DENV1.
